# When the disposition effect proves to be rational: Experimental evidence from professional traders

**DOI:** 10.3389/fpsyg.2023.1091922

**Published:** 2023-02-23

**Authors:** Benno Guenther, Grace Lordan

**Affiliations:** Department of Psychological and Behavioural Science, London School of Economics and Political Science, London, United Kingdom

**Keywords:** disposition effect, prospect theory, commodities, behavioural finance, trading decision-making JEL classification: G11, G41

## Abstract

The disposition effect is a behavioural finance anomaly that has been observed in many populations including non-professional investors as well as professional investors and has been linked to reduced trading performance. However, the majority of studies to date have looked at the disposition effect in the context of non-mean reverting markets. We conducted a within-subject experiment with *n* = 193 professional traders, to examine how the tendency towards the disposition effect varies across decision-making for mean reverting securities and non-mean reverting securities. In addition, we consider whether a simple informational intervention that makes the disposition effect salient can alter decision-making. Overall, we find that prior to the intervention the traders exhibit the disposition effect in the direction that aligns with profit maximisation goals suggesting that they are acting rational. For decisions on mean reverting securities the traders tend to make decisions in the direction of the disposition effect, which is rational given their mean reverting properties. We also find that the informational intervention is effective in changing the level of the disposition effect observed and decision-making, regardless of whether traders are considering decisions over mean reverting or non-mean reverting securities. Further, we provide evidence that our simple informational intervention improves trader returns when making decisions on non-mean reverting securities. In contrast, it has a negative impact when utilised for mean reverting securities. Our study highlights the power of simple interventions to make disproportionately large changes to decision-making regardless of whether they are in our best interests, and their beneficial role only when the context is right.

## Introduction

The disposition effect, or the tendency for investors to close out winning positions faster than losing ones, is a behavioural finance anomaly, observed in many populations including non-professional investors (Odean, 1998; Grinblatt and Keloharju, 2001; Shapira and Venezia, 2001; Dhar and Zhu, 2002; Barber et al., 2007; Chen et al., 2007; Hmida and Boussaidi, 2017; Shandu and Alagidede, 2022) as well as professional investors (Ferris et al., 1988; Coval and Moskowitz, 1999; Shapira and Venezia, 2001; Garvey and Murphy, 2004; Frazzini, 2006; Barber et al., 2007; Wulfmeyer, 2016) and is owed to Shefrin and Statman (1985). This anomaly has been linked to lower trading performance (Odean, 1998; Garvey and Murphy, 2004; Aspara and Hoffmann, 2015; Koestner et al., 2017) and momentum in stock markets (Grinblatt and Han, 2005), highlighting it as an important phenomenon with implications for an individual’s investment returns and financial markets as a whole (Coval and Shumway, 2005).

Biases, like the disposition effect, are also of interest to those who manage traders in investment banks or hedge funds. For example, to the extent that the disposition effect is causing lower levels of performance among team members, managers may choose to explore behavioural interventions to mitigate it. However, this highlights the importance of determining first whether the disposition effect is indeed present in the population the manager is interested in, and also determining whether any effects found impact returns. It stands to reason that the presence of the disposition effect can be problematic for equity traders, and performance augmenting for commodity traders. This arises because commodities are securities that tend to mean revert (Andersson, 2007; Lawal et al., 2019), whereas equities on average do not. Therefore, in certain contexts exhibiting the disposition effect can in fact prove to be beneficial. This nuanced understanding has not yet come through in the literature and implies that exhibiting the disposition effect can be rational in certain contexts. In addition, the literature is notably lacking on studies that examine the extent to which behavioural interventions can mitigate the disposition effect, and how effects on returns differ over short, medium, and long horizons depending on whether the context is mean reverting or non-mean reverting. This is an important consideration for managers in finance. In this study we make two distinct contributions to the literature:

First, we test whether the disposition effect exists in a population of professional traders *via* an online within-subject experiment, where the large majority are commodities traders. We separate the effects found by mean reverting and non-mean reverting securities^1^ and demonstrate how they impact performance over the short (5 week), medium (6 months) and long run (12 months). Following Odean (1998), the disposition effect is measured for each participant as the difference between the proportion of gains realised (PGR) and the proportion of losses realised (PLR). There are two main reasons that the disposition effect frequently found in the literature in student and other adult populations, has been documented to a lesser degree for more sophisticated investors (Dhar and Zhu, 2006) as well as more experienced investors (Chen et al., 2007). First, professional traders learn by doing, and arguably over time only those that weed out biases that negatively impact their performance should survive. Profit and loss is after all an objective statement of performance over the medium term when luck runs out. Therefore, we may expect that professional traders will have a tendency to exhibit the disposition effect when it is to their benefit, i.e., they are trading mean reverting securities such as commodities. Conversely, we may expect them to exhibit the reverse disposition effect, i.e., closing losing positions faster than winning positions, when trading non-mean reverting securities such as equities. This underlines the importance of separating the disposition effect by mean reverting and non-mean reverting securities as we do in this study, as well as considering multiple investment horizons. Second, professional traders are less exposed to emotional attachment to the money they are investing. This may, for example, cause them to succumb less to the pain of loss aversion and hold losing stocks too long to claw their losses back feeding the disposition effect. Overall, we therefore expect that the disposition effect is lower or zero for professional traders, or moves in a direction that is rational (i.e., money making). We note that in cases where traders are beholden to their clients, the disposition effect may still be observed if clients press them to unwind winning positions too early even if the traders do not exhibit the disposition effect. Therefore, in our work we choose to include only market makers, so that the constraints of following client wishes are not ingrained habits.

Second, this study is, to the best of our knowledge, the first experimental study conducted with professional traders to test the impact of a behavioural intervention in the context of the disposition effect. Previous studies suggest that the disposition effect is detrimental to trading performance (Odean, 1998; Aspara and Hoffmann, 2015; Duxbury et al., 2015; Shen and Shen, 2022) and imply that market participants would benefit from not displaying the disposition effect or even displaying the reverse disposition effect. Given that these studies relate to securities that do not revert to the mean on average, drawing this conclusion makes sense. Therefore, our work overlaps and extends what has gone before by examining whether a simple behavioural intervention changes the disposition effect, and how it impacts short, medium and long run performance separately by mean reverting and non-mean reverting positions. This approach allows us to directly quantify the benefit of the intervention over three time horizons, and also allows us to see if professional traders are less likely to be influenced by a behavioural intervention that is not in their interests (i.e., when they are making decisions on mean reverting securities).

The background of this study is related to a growing body of research into the susceptibility of financial decisions to biases (Fisher and Statman, 2000; Wulfmeyer, 2016). Differences in how people emotionally experience and cognitively assess risk (Kahneman and Tversky, 1979) offers a potential explanation for these deviations from efficient capital market theory (Fama, 1970). While there is no overall consensus on the underlying reasons or explanation for the existence of the disposition effect, a combination of prospect theory (Kahneman and Tversky, 1979; Tversky and Kahneman, 1992) and mental accounting (Thaler, 1985) has emerged as the leading explanation (Grinblatt and Han, 2005; Meng, 2011; Summers and Duxbury, 2012). Mental accounting can help understand how people assess individual portfolio positions as separate (mental) accounts rather than one account or portfolio. Prospect theory is a descriptive model developed by Kahneman and Tversky (1979) for choice under uncertainty and describes the higher propensity to hold on to losing positions relative to winning ones. Its central point is that individuals make decisions based on their experienced value, measured as losses and gains, relative to a fixed reference point. This is in contrast to standard expected utility theory as introduced by von Neumann and Morgenstern (1944) where utility is generally calculated based on terminal levels. Moreover, prospect theory has an s-shaped value function: being concave in the gain domain and convex in the loss domain describing risk-aversion and risk-seekingness, respectively (Grinblatt and Han, 2005). Additionally, the value function is steeper for losses than it is for gains, indicating loss-aversion.

We also note several studies questioning the role of prospect theory in the context of the disposition effect. One of the criticisms is its descriptive nature: while it *describes* how people make choices under uncertainty, it does not *explain* why they do so as emotions are not included in the decision-making process (Sunstein, 2002). Most notably Summers and Duxbury (2012) suggest that prospect theory is not sufficient to explain the disposition effect, and suggest that regret and elation are necessary causes of the disposition effect. Additional explanations include cognitive dissonance (Hartzmark and Solomon, 2010; Chang et al., 2016) and a (rational or irrational) belief in mean reversion (Barberis and Thaler, 2003; Jiao, 2017). Using functional magnetic resonance imaging (fMRI), Brooks et al. (2012) conclude that the existence of the disposition effect is most consistent with a belief in mean reversion.

Our work also relates to studies that have looked for empirical evidence of the disposition effect in investors drawing on observational data. For example, Odean (1998) analysed trading information from brokerage accounts of 10,000 households between 1987 and 1993 and highlights the presence of the disposition effect, emphasising that this causes returns to be 4.4 percent lower *per annum*. Dhar and Zhu (2002) examined trading records from a large discount brokerage of about 50,000 individual investors between 1991 and 1996. They found evidence for the existence of the disposition effect on both aggregate and individual investor level. Suggestive evidence of the disposition effect has been found in many different countries and cultures including China (Chen et al., 2007), Finland (Grinblatt and Keloharju, 2001), Israel (Shapira and Venezia, 2001), South Africa (Shandu and Alagidede, 2022), Taiwan (Shu et al., 2005; Barber et al., 2007), and Tunisia (Hmida and Boussaidi, 2017). The disposition to sell winners as opposed to losers is also not limited to private investors. A number of studies found evidence of the disposition effect amongst professional investors when trading equities (Ferris et al., 1988; Shapira and Venezia, 2001; Garvey and Murphy, 2004; Barber et al., 2007) and mutual fund managers (Coval and Moskowitz, 1999; Frazzini, 2006; Wulfmeyer, 2016). One of the few published papers on the disposition effect in the context of short positions (see von Beschwitz and Massa (2015)) investigated a dataset of stock lending for all U.S. stocks between 2004 and 2010 and concluded that short sellers also exhibit the disposition effect. In our work we randomly assign the decisions faced by the traders to be a combination of short versus long positions, to increase the external validity of our conclusions.

Moreover, our work directly relates to lab experiments that look for the disposition effect in both naïve and sophisticated investors. Most relevant are those studies that consider whether an intervention can alter the disposition effect. The first known study is owed to Weber and Camerer (1998) who worked with students in a lab experiment over 14 trading periods asking their participants to make hypothetical buy or sell (but not short sell) decisions across six risky assets. Overall, 60% of the shares sold are winning shares versus 40% losing, hence finding support for the disposition effect. However, when shares were automatically (by default) sold at the end of each period, the authors found a significant reduction in the disposition effect. While defaults or pre-selected choices have generally been found to be very effective, there is significant variation in a default’s effectiveness based on the respective domain (Jachimowicz et al., 2019). We also note, that although effective, it is difficult to imagine how this specific intervention could practically be implemented on a trading floor. Ploner (2017) conducted a between-subjects lab experiment with 159 mostly student participants and found evidence for the existence of the disposition effect in a simple trading simulation. Moreover, he observed the reverse disposition effect for the treatment group, which was asked to decide ahead whether they would like to keep or close out the position depending on the next move of the stock. This plan was binding and notably has some resemblance to limit orders which also have been shown to reduce the disposition effect (Fischbacher et al., 2017). In one of their experimental studies, Lee et al. (2008) found that the disposition effect can be reduced when individuals are instructed to imagine they are investing for someone else. Their findings are consistent with other studies that document that the magnitude of the disposition effect depends on whether individuals own a stock through their own choice or not (Summers and Duxbury, 2012) and on feelings of personal responsibility (Aspara and Hoffmann, 2015).

In another stock trading experiment conducted by Frydman and Rangel (2014) with 58 Caltech students, participants were given an initial amount of virtual money to trade in three different stocks and incentivised to maximise the portfolio return. The students were then randomly assigned to either the *high-saliency* condition or the *low-saliency* condition. While Frydman and Rangel (2014) find the disposition effect in both conditions, the disposition effect in the low-salience group is 25% lower, suggesting that the disposition effect can be mitigated by reducing the salience of the stock purchase price. Dobrich et al. (2014) conducted an online experiment with 223 private investors. In this study the authors used a between subject design where participants were randomly allocated to one of four conditions: (1) no intervention, (2) rational debiasing intervention, (3) emotional debiasing intervention or (4) combined rational and emotional debiasing intervention. While they found a significant average disposition effect in the control condition, they also found a significant average *reverse* disposition effect in all three treatment conditions, indicating that their interventions successfully mitigated the disposition effect. It is noteworthy that these are two examples of simple interventions, that can be readily deployed to alter the behaviour of professional investors, once the disposition effect’s impact on returns over various investment horizons has been considered. This is in the same spirit of our intervention, which can be easily adopted by traders to help their day-to-day decision-making.

Drawing on a within-subject experiment that involved 193 professional traders, with 151 commodities traders, we demonstrate that traders exhibit the disposition effect for decisions regarding mean reverting securities, and the reverse disposition effect for decisions regarding non-mean reverting securities prior to any informational intervention. This suggests that traders are on average rational as these tendencies align with strategies for profit maximisation. This compares to research on non-professional populations that highlights the disposition effect when making choices over non-mean reverting securities (Odean, 1998; Dhar and Zhu, 2002). In addition, we demonstrate that a simple informational intervention which makes the tendency for the disposition effect salient, is effective in changing decision-making on average. It is noteworthy, that our intervention blatantly gives information to traders without constraining their choice set, thus giving them full autonomy over their decision-making. It is also noteworthy that the intervention is effective regardless of whether traders are considering choices on mean reverting or non-mean reverting securities. This underlines the importance of considering that interventions are indeed fit for purpose and monitoring their success, as in the mean reverting setting (commodities) we highlight that the intervention would negatively impact returns in the short and medium term. However, in the mean reverting setting performance is improved across all three time horizons that we consider, emphasising that simple interventions are worthwhile when the context is right.

## The experiment

By conducting a within-subject experiment this study examines the disposition effect in the context of professional (commodity) traders as well as the impact of an informational intervention. Data was collected in an online Qualtrics based experiment between August 2018 and October 2018 from *N* = 193 participants with self-reported professional trading experience ranging from 0.6 to 30 years (*M* = 8.6, *SD* = 5.8). One author with a significant personal network of professional traders recruited all participants *via* direct messages on LinkedIn. The experiment was conducted in accordance with the research ethics policy and procedures of the authors’ home institution. Out of all participants 92.2% indicated being male^2^ and 78.2% reported trading commodities with average self-reported trading experience of 8.46 years (*SD* = 5.92) for male participants and 8.25 years (*SD* = 5.36) for commodity traders. We performed an ex-ante sample size calculation using R based on an estimated standardised mean difference for our informational intervention of *d* = 0.4 at the 5% significance level and 90% power, resulting in a required sample size of *n* = 133 participants per condition.

After giving their informed consent and viewing the experimental instructions, the participants were shown a series of ten candlestick charts^3^ of actual tradeable securities covering 50 trading periods each as shown in Figure A1. In addition to seeing the security chart, participants are told for each security that they had entered the position at a given price (fixed by security across all participants) and whether their position was a long or a short position (randomly assigned with equal probability for every security and every participant). Moreover, participants are informed about the last traded price of the security (fixed by security across all participants) and are asked whether they would like to keep the position (selected as a default^4^) or whether they would like to close out their position at the last traded price. While the data we use to create the candlestick charts is based on periods of 1 week, this information is not disclosed to the participants who are asked to make their decisions to maximise their performance over the next five trading periods. This allows each trader to work to their preferred time horizon, which we assume they will in the absence of a specified time frame. In our study the participants were incentivised to participate and maximise their trading performance over 5 periods starting from their decision by awarding the highest portfolio performance with GBP 250 as well as informing participants about their individual trading performance relative to their peers.

The ten securities are grouped into two blocks of five securities each and the order of these two blocks is randomly assigned for every participant.^5^ After half of the trading decisions (before the sixth exhibit), all participants were shown an informational intervention about the existence of the disposition effect similar to Dobrich et al. (2014). The first five questions displayed to each participant before the intervention form the basis of the *control condition* and the five questions after the interventions form the basis of the *treatment condition*. More specifically, the following text was displayed to all participants between the fifth and sixth exhibit:

### Information for maximising your investment performance

*Investors typically fall prey to two performance reducing investment flaws:*

1. *Closing out winning positions too early (due to the experience of pride of a right decision)*
2. *Holding losing positions for too long (avoiding regret that an investment decision was wrong).*

*However, previous studies indicate that it is generally advantageous to hold winning positions and to limit losses by closing losing positions. You can, for instance, think of specific thresholds at which you close a specific position.*

It is noteworthy that this informational intervention blatantly gives information to traders and does not constrain their choice set giving them full autonomy over their decision-making. As mentioned previously, the order of the questions before and after the intervention were randomly assigned to balance the within-subject design, as was whether a participant was faced a decision on a long and short position.

Based on the decisions made by the individual participants, we calculate individual profit and loss over a short (5 weeks), medium (6 months) and long (1 year) horizon. The first stage of the experiment (control) allowed us to establish a baseline performance in the absence of a behavioural intervention, and to consider how any disposition effects exhibited by the traders in our study affects their performance over 5 weeks, 6 months, and 12 months. Full details on instructions given in first stage can be found in Appendix A.

A description of the securities used in this study is provided in Table 1. We define an underlying (from herein we call security) as mean reverting if the sign of the return between the position opening and the decision is different from the sign of the return between the decision and the price 5 weeks after the decision. Otherwise, the underlying is classified as non-mean reverting. Based on this definition six securities are mean reverting and four are non-mean reverting.

**Table 1:** Securities depicted in choice sets.

Underlying	Description	Unit	Asset class	Mean reverting?
EURUSD	Euro – US Dollar Exchange Rate	US Dollar per Euro	Foreign Exchange	No
Brent Z8	Brent Crude December 2018 Future	US Dollar per Barrel	Commodity	Yes
SPX TR	S&P 500 Total Return Index	US Dollar	Equity	No
XAUUSD	Gold Spot Price	US Dollar per Troy Ounce	Foreign Exchange	Yes
Tesla	Tesla Inc. Share Price	US Dollar per Share	Equity	Yes
US NatGas Z8	Henry Hub Natural Gas December 2018 Future	US Dollar per MMBtu	Commodity	Yes
JPYUSD	Japanese Yen – US Dollar Exchange Rate	US Dollar per Yen	Foreign Exchange	Yes
Coffee Z8	Arabica Coffee December 2018 Future	US Dollar per Pound	Commodity	No
BTCUSD	Bitcoin Spot Price	US Dollar per Bitcoin	Cryptocurrency	Yes
Wheat Z8	Wheat December 2018 Future	US Dollar per Bushel	Commodity	No

An underlying is classified as mean reverting throughout this study if and only if the sign of the return between the position opening and the decision is different from the sign of the return between the decision and the price 5 weeks after the decision; otherwise the underlying is classified as non-mean reverting.

### Measuring the disposition effect

This study uses the method of Odean (1998) where the disposition effect (DE) is calculated as the difference of the Proportion of Gains Realised (PGR) and the Proportion of Losses Realised (PLR). More formally these are defined as:


(1) $$PGR=\frac{numberofrealisedgains}{numberoftotalgains}$$

(2) $$PLR=\frac{numberofrealisedlosses}{numberoftotallosses}$$

(3) DE=PGR−PLR

The main variable of interest for this study is the difference between the proportion of gains realised and the proportion of losses realised (*DE PGR PLR* = −). By construction this measure can only assume values between −1 and +1 and is a continuous variable on this interval. Moreover, there are three distinct cases of interest:

1. The measure is larger than zero in which case we observe the disposition effect
2. The measure equals zero in which case we do not observe the disposition effect
3. The measure is smaller than zero in which case we observe the reverse disposition effect.

Equations (1) through (3) are calculated separately for each stage of the experiment (control and treatment), as well as by whether a security is mean reverting or non-mean reverting.^6^

**Figure 1:**
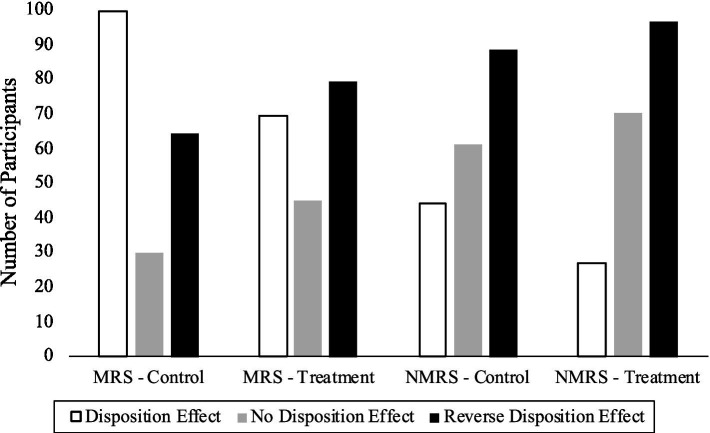
Number of traders exhibiting the disposition effect, no disposition effect, and the reverse disposition effect for mean reverting securities (MRS) and non-mean reverting securities (NMRS) across the control and treatment condition.

It follows that when considering mean reverting long and short positions a rational trader would have Eq. (3) values ranging from zero to plus one, whereas a rational trader considering non-mean reverting exhibits would have values ranging from zero to minus one. Turning to Figure 1 it is noted that this does hold true on average in our data. That is, if we consider mean reverting securities, in the control phase (or phase 1) many more traders exhibit the disposition effect when making decisions on mean reverting securities (99), as compared to when making decisions on non-mean reverting securities (44). This suggest that exhibiting the disposition effect is endogenous to the type of security being considered, and hints at conscious or unconscious switching based on the data being presented. We note the opposite pattern for the reverse disposition effect. That is, it occurs more often on decisions regarding non-mean reverting securities. Still, Figure 1 highlights that 44 traders do succumb to the disposition effect when making decisions on non-mean reverting securities, a significant number of individuals (23%) whose performance can potentially be improved by a simple intervention that reduces this tendency.

Figure 1 also documents the number of traders that exhibit the disposition effect after the informational intervention (treatment). It is noteworthy, that this intervention looks to be always effective, regardless of whether this change is of benefit (i.e., the decision is being made on a non-mean reverting security where incidence falls from 44 to 27), or not (i.e., the decision is being made on a mean reverting security where incidence falls from 99 to 69).

To examine more completely how the tendency to exhibit the disposition effect differs across mean-reverting and non-mean reverting securities, as well as before and after our informational intervention we follow Dobrich et al. (2014) and estimate:


(4) $$\;DE_{i}^{C}=\beta_{0}+\varepsilon_{i},$$

(5) $$\;DE_{i}^{T}=\beta_{0}+\varepsilon_{i}\;.$$

In Eqs (4) and (5) $DE_{i}^{C}$ and $DE_{i}^{T}$ are calculated according to Eq. (3) for the control phase and the treatment phase, respectively.^7^ Estimating Eqs (4) and (5) gives the benefit of allowing us to quantify the intensity of the disposition effect. That is, the intercept of this regression represents the average disposition effect displayed by the participants. We probe for heterogenous treatment effects by estimating Eqs (4) and (5) separately for males (we do not have adequate sample to consider a female only analysis), and tenure (splitting the sample by its median into tenure that is longer than 7 years and less than or equal to 7 years to ensure approximately equal size). We may expect traders with longer tenure to exhibit greater rationality, assuming those with unhelpful biases that impact trading performance do not survive in the long run.

We wish to consider the impact of our informational intervention explicitly on returns potential. We hypothesise that it will be beneficial for decisions regarding non-mean reverting securities, and detrimental for those regarding mean reverting securities. To consider this we calculate returns separately for the control and treatment phase (phase 1 and phase 2 respectively). For each participant, we calculate the ex-post return for every security over 5 weeks, 6 months and 1 year, where the return is zero if the participant decided to close out the position. Otherwise, in the case of a long position the return equals the return of the security and in the case of a short position negative the return of said security. We then group the securities for each tenor (5 weeks, 6 months and 1 year) in a 2 × 2 fashion: control vs. treatment and mean reverting vs. non-mean reverting. For each of these categories, we calculate the average return by participant. Appendix C documents the mean returns resulting from these calculations for each security separately for 5 weeks, 6 months and 1 year.

## Results

Table 2 documents the estimates from Eqs (4) and (5). Three stylised facts emerge. First, in the control phase, traders exhibit the disposition effect for mean reverting securities, suggesting they are on average rational. Second, the same group of traders switch to displaying on average the reverse disposition effect when making decisions on non-mean reverting securities, again suggesting that the traders are rational. Third, the informational intervention is effective in changing behaviour for decisions regarding both security types to approximately the same extent. This serves to cause traders making decisions on mean reverting securities to no longer exhibit the disposition effect, and to increase the reverse disposition effect as compared to the control phase when the traders are making decisions regarding non-mean reverting securities. An ex-post power calculation (*d* = 0.32, alpha = 0.05 and *n* = 193) yields a power of 0.88. While the results reported are based on the calculation of the disposition effect according to Odean (1998), the results replicate using the alternative calculation procedure according to Weber and Camerer (1998).^8^

**Table 2:** Main estimates.

	Mean reverting		Non-mean reverting securities	
	DE^C^	DE^T^	DE^C^	DE^T^
Intercept	0.1529*** (0.0428)	−0.0380 (0.0428)	−0.2047*** (0.0493)	−0.3601*** (0.0449)
Num. obs.	193	193	193	193

Estimates from Eqs (4) and (5). DE^C^ and DE^T^ represent the disposition effect for the control group and treatment group, respectively, calculated following Eqs (1) through (3).

Table 3 documents sub-group analysis by gender and tenure. Specifically, Table 3 indicates that the pattern found in Table 2 is marginally more pronounced when we restrict our regressions to males only. Table 3 also shows that traders with more years of tenure do not exhibit any significant disposition effect in the control phase, and do not respond to our intervention when they are making decisions on mean reverting securities. In other words, they do not follow the intervention when the resulting effect on their performance is predicted to be negative ex ante. However, they do respond when securities are non-mean reverting in a direction that likely positively impacts their returns. In contrast, those with 7 years or less experience do display a significant disposition effect when making decisions on mean reverting securities, and a significant reverse disposition effect when making decisions on non-mean reverting securities. This group of participants always respond significantly to the informational intervention.

**Table 3:** Sub-group analysis.

Mean reverting securities						
	Male only		Experience >7 years		Experience ≤7 years	
	DE^C^	DE^T^	DE^C^	DE^T^	DE^C^	DE^T^
**Intercept**	0.1817*** (0.0441)	−0.0618 (0.0437)	0.1083 (0.0581)	−0.0653 (0.0561)	0.1979** (0.0628)	−0.0104 (0.0649)
**Num. obs.**	178	178	97	97	96	96
**Non-mean reverting securities**						
	**Male only**		**Experience >7 years**		**Experience ≤7 years**	
	DE^C^	DE^T^	DE^C^	DE^T^	DE^C^	DE^T^
**Intercept**	−0.2079*** (0.0514)	−0.3736*** (0.0461)	−0.1340 (0.0693)	−0.3299*** (0.0663)	−0.2760*** (0.0698)	−0.3906*** (0.0607)
**Num. obs.**	178	178	97	97	96	96

Estimates from Eqs (4) and (5). DE^C^ and DE^T^ represent the disposition effect from the control group and treatment group, respectively, calculated following Eqs (1) through (3).

To explore the impact on returns, Table 4 documents returns separately for bundles of mean reverting and non-mean reverting securities by the control and treatment phases, separately for the full sample, males only, and tenure length. We note that the returns for the mean reverting securities are lower in the treatment as compared to the control group over the short (5 weeks) and medium term (26 weeks) but augmented over the long run (52 weeks). Recall, that on average our informational intervention served to decrease the intensity of the disposition effect. This suggests that the value of exhibiting the disposition effect (with high levels of intensity) is related to the horizon of the investment in mean reverting securities, and in the case of our selection of securities it is valuable when investors have a long run horizon (compared to the mean reversion cycle). We note though, that it does imply that our informational intervention is detrimental for investors with short-or medium-term horizons, as we expected. In contrast, our informational intervention is always beneficial, regardless of the time horizon when decisions are being made on non-mean reverting securities. This also aligns with our expectations, and notably the information intervention allows for better returns across both levels of experience we consider.

**Table 4:** Returns that would have been realised based on the hypothetical decisions made.

	Full sample		Males		>7 years experience		≤7 years experience	
	Mean reverting	Non-mean reverting	Mean reverting	Non-mean reverting	Mean reverting	Non-mean reverting	Mean reverting	Non-mean reverting
**5 week returns**								
Control	0.20%	0.30%	0.22%	0.30%	0.02%	0.10%	0.38%	0.49%
Treatment	−0.60%	0.93%	−0.67%	0.96%	−0.71%	1.02%	−0.49%	0.84%
**6 month returns**								
Control	−1.23%	−0.26%	−1.20%	−0.15%	−1.04%	−0.39%	−1.42%	−0.12%
Treatment	−3.30%	0.05%	−3.46%	0.09%	−3.97%	0.28%	−2.63%	−0.18%
**12 month returns**								
Control	−0.69%	−0.13%	−0.81%	0.07%	0.06%	−0.39%	−1.46%	0.13%
Treatment	2.17%	0.69%	2.39%	0.69%	2.59%	0.67%	1.75%	0.72%

For each participant, we calculate the post-decision return for every security over 5, 26 and 52 weeks, where the return is zero if the participant decided to close out the position. Otherwise, in the case of a long position the return equals the return of the security and in the case of a short position negative the return of said security. We then group the securities for each tenor (5, 26 and 52 weeks) in a 2 × 2 fashion: control vs treatment and mean reverting vs non-mean reverting. For each of these categories, we calculate the average return by participant. Appendix D documents the mean returns resulting from these calculations for each security separately by 5 weeks, 6 months and 1 year.

While we note that our study depicted an overall downward market trend which might have impacted the results, we assigned long and short positions randomly for each participant and position, thus removing this trend from the aggregate portfolio profit and loss account. Moreover, the measure of Odean (1998) controls to some extent for the potential impact of the market trends and is regarded as an unbiased calculation method for the disposition effect (Corneille et al., 2018).

## Conclusion

This study utilises a within-subject experiment with 193 professional traders, 151 of which are commodity traders, to examine how the tendency towards the disposition effect varies across decision-making in mean reverting (e.g., commodities) and non-mean reverting (e.g., equities) contexts. In addition, we consider how an informational intervention that makes the disposition effect salient changes the decision making of these participants across these two contexts. Three stylised facts emerge from our analyses. First, prior to the informational intervention the traders in our study demonstrate the disposition effect for decisions regarding mean reverting securities, and the reverse disposition effect for decisions regarding non-mean reverting securities. This suggests a degree of rationality as these tendencies align with profit maximisation goals.

Second, the informational intervention is effective in changing decisions regardless of whether traders are considering decisions over mean reverting or non-mean reverting securities. The change in the disposition effect is also of the same order of magnitude for mean reverting and non-mean everting securities. In other words, it is profit enhancing for equities traders, but damaging for commodities traders.

Third, the informational intervention would have positively affected the returns over a 5 week, 6 months or 1-year horizon if the traders’ decisions on non-mean reverting securities were realised. In contrast, it would have negatively impacted the returns for mean reverting securities if realised over 5 weeks or 6 months. This highlights the importance of the context as well as the power of simple interventions to make disproportionate changes to our decision-making.

Overall, we view the results of this intervention as very promising with regards to its ability to improve professional trader’s performance in the right context. Notably, and intentionally, our intervention blatantly gives information to traders, and does not constrain their choice set giving them full autonomy over their decision-making. Future research could further improve on the research design and generalisability, by moving from a hypothetical within subject design, to a randomised control trial in the field focused only on equity traders. This allows for an intervention to be trialled that is expected to benefit participants ex ante, in a real world setting with high stakes decisions that will move our learning forward by allowing us to consider both adaptation and efficacy.

## Data availability statement

The raw data supporting the conclusions of this article will be made available by the authors, without undue reservation upon request.

## Ethics statement

The studies involving human participants were reviewed and approved by London School of Economics. The patients/participants provided their written informed consent to participate in this study.

## Author contributions

BG conceived the experiment under the guidance of GL, who advised on appropriate design, data collection and methodology. BG drafted the first draft including the literature review. GL edited and refined the draft with BG. All authors contributed to the article and approved the submitted version.

## Funding

LSE will fund publication.

## Conflict of interest

The authors declare that the research was conducted in the absence of any commercial or financial relationships that could be construed as a potential conflict of interest.

## Publisher’s note

All claims expressed in this article are solely those of the authors and do not necessarily represent those of their affiliated organizations, or those of the publisher, the editors and the reviewers. Any product that may be evaluated in this article, or claim that may be made by its manufacturer, is not guaranteed or endorsed by the publisher.

## Footnotes

^1^Throughout this study a security is classified as mean reverting if and only if the sign of the return between the position opening and the decision is different from the sign of the return between the decision and the price 5 weeks after the decision; otherwise the underlying is classified as non-mean reverting.

^2^The low number of female participants is similar to Coates (2012) who mentions that women constitute about 5% of trading floors.

^3^Candlestick charts are used widely by professional traders for historical price visualisation (Menkhoff, 2010) and contain valuable information about recent trends, ranges and volatility levels. An example of the chart display can be found in Appendix A.

^4^While this is purposely chosen as the default to mimic real-word trading situations where a position is kept (by default) unless action is tacking to close it out, we note that the option to hold onto the position represents the riskier option, which has shown to promote a larger default effect (Giuliani et al., 2022).

^5^The first block consists of the first five securities in Table 1 i.e., EURUSD, Brent Z8, SPX TR, XAUUSD and Tesla. The second block is comprised of the remaining securities, i.e., US NatGas Z8, JPYUSD, Coffee Z8, BTCUSD and Wheat Z8.

^6^The 193 participants made a total of 1,930 trading decisions in the study that form the basis of the statistical analysis. Each experimental phase (control and treatment) consists of five decisions to be made by every participant, within each phase there are three mean reverting (1,158 trading decisions in total across phases) and two non-mean reverting securities (772 trading decisions in total across phases).

^7^In Appendix B we also document estimates from these equations that control for gender and years of tenure, which do not change the conclusions drawn in this work.

^8^Weber and Camerer (1998) define the disposition effect as the difference between the number of sold winning securities $S_{+}$ and the number of sold losing securitiesties ($S_{-}$, divided by the total number of sold securities, or more formally: $DE_{WC}=\frac{S_{+}-S_{-}}{S_{+}+S_{-}}$.
