# Supplementary material for: When the disposition effect proves to be rational: Experimental evidence from professional traders
Source: Front Psychol. 2023 Feb 23;14:1091922. doi: 10.3389/fpsyg.2023.1091922 (PMC9996105; doi:10.3389/fpsyg.2023.1091922)
Supplement: Supplementary file 1 [file Data_Sheet_1.docx]

# Appendix A: Experiment Instructions

On the following pages you will be shown ten candlestick charts of tradeable securities. These ten securities form part of your current portfolio with an equal notional investment into each position of $1,000,000. Your portfolio will be evaluated five trading periods in the future and your task is to maximise your portfolio performance. The participant with the highest portfolio performance will receive a retail voucher worth GBP 250 (or the equivalent in your local currency). As participant you can also opt to be notified about your portfolio performance and how your performance compares to your peers.

For each position you will be provided with a candlestick chart for the 50 previous trading periods for that security (based on actual tradeable security data), the level at which the position was entered and if your position is a long or a short position. You will then be asked to decide for each individual security whether you want to keep the position open or close it out.

Good luck and remember your task is to maximise the portfolio performance five trading periods from now!

# Figure A.1: Sample security chart.


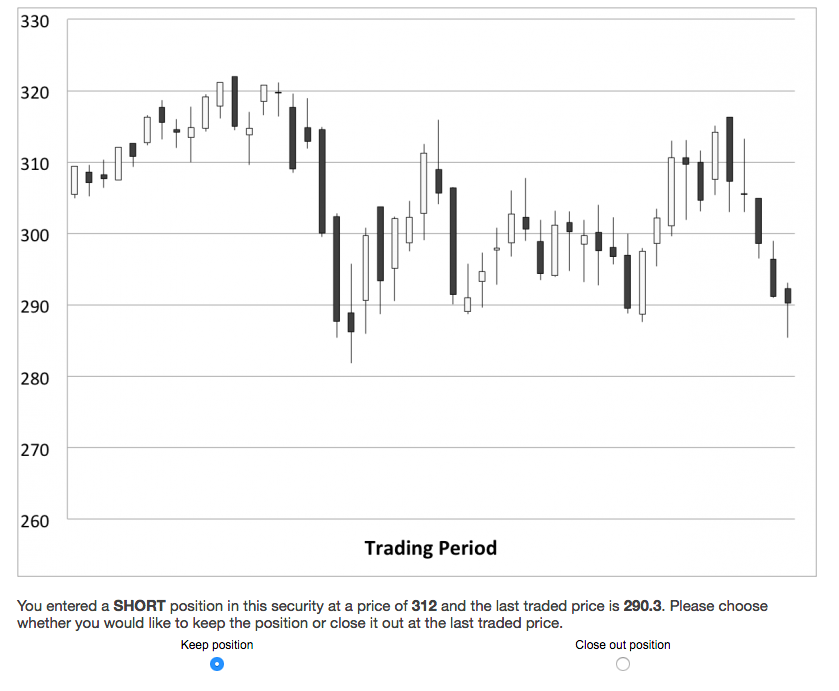


Note: All security prices are in USD and rebased to arbitrary prices to eliminate the participant’s possibility to second-guess the underlying security (identical scalar transformation for each security across participants). Positions are randomly assigned to be long or short for every security and participant (each participant on average sees five long and five short positions) at the opening price, which is fixed for each security across participants. ‘Keep position’ is selected as default for each of the ten securities just as it is the default in a trading situation.

**Appendix B: Robustness Analysis**

Table B.1: Mean reverting securities only for all participants.

|  | Mean Reverting Securities | | Non-Mean Reverting Securities | |
| --- | --- | --- | --- | --- |
|  | **DE^C^** | **DE^T^** | **DE^C^** | **DE^T^** |
| Intercept | 0.1979*  (0.0785) | 0.0031  (0.0766) | -0.2429**  (0.0914) | -0.3204***  (0.0816) |
|  |  |  |  |  |
| Female | -0.3582  (0.2165) | 0.2225  (0.2111) | 0.0731  (0.2520) | 0.1383  (0.2250) |
|  |  |  |  |  |
| Experience | -0.0028  (0.0076) | -0.0082  (0.0074) | 0.0046  (0.0088) | -0.0070  (0.0079) |
| Num. obs. | 182 | 182 | 182 | 182 |

**Appendix C: Mean returns for 5 weeks, 6 months and 1 year time horizons.**

The tables below summarise the return of individual securities pre and post the trading decision and their respective contribution to the mean-reversion of the investment universe. There are three tables with the corresponding values for post-decision periods of 5, 26 and 52 weeks respectively. The pre-decision return is the return of the security between the position opening price and the security price when participants make the decision whether to keep the security or close it out. The post-decision return is the return of the security between the security price when participants make the decision whether to keep the security or close it out and the closing price 5, 26 or 52 weeks later. The mean reversion contribution is the absolute value of the security’s post-decision return divided by the sum of the absolute post-decision returns of all mean reverting securities.

All commodity underlyings (except for gold) are based on fixed expiry December 2018 future contracts. As we expand the analysis of the returns beyond the five-week horizon, all these futures have been ‘rolled’ into the corresponding December 2019 future contract based on the closing prices of 24^th^ August 2018 which marks the end of the five week period.

Table C.1: 5 week returns.

| **Underlying** | **Classification** | **Pre-Decision Return** | **Post-Decision Return** | **Absolute Post-Decision Return** | **Mean reverting** | **MR contribution** |
| --- | --- | --- | --- | --- | --- | --- |
| **EURUSD** | CCY | -5.57% | -0.87% | 0.87% | 0 | 0% |
| **Brent Z8** | Commod | -7.55% | 3.89% | 3.89% | 1 | 16% |
| **SPX TR** | Index | 9.53% | 2.80% | 2.80% | 0 | 0% |
| **XAUUSD** | Commod | 8.47% | -1.97% | 1.97% | 1 | 8% |
| **Tesla** | Stock | -3.77% | 2.95% | 2.95% | 1 | 12% |
| **US NatGas Z8** | Commod | -6.96% | 4.79% | 4.79% | 1 | 20% |
| **JPYUSD** | CCY | -4.76% | 0.19% | 0.19% | 1 | 1% |
| **Coffee Z8** | Commod | -18.78% | -8.20% | 8.20% | 0 | 0% |
| **BTCUSD** | Crypto | 21.43% | -10.06% | 10.06% | 1 | 42% |
| **Wheat Z8** | Commod | 11.39% | 0.66% | 0.66% | 0 | 0% |
| **Total** |  | 3.43% | -5.82% |  | 6 | 100% |
| **Correlation** |  | **-20.91%** |  |  |  |  |

Table C.2: 6 month returns.

| **Underlying** | **Classification** | **Pre-Decision Return** | **Post-Decision Return** | **Absolute Post-Decision Return** | **Mean reverting** | **MR contribution** |
| --- | --- | --- | --- | --- | --- | --- |
| **EURUSD** | CCY | -5.57% | -3.08% | 3.08% | 0 | 0% |
| **Brent Z8 (Z9)** | Commod | -7.55% | -11.35% | 11.35% | 0 | 0% |
| **SPX TR** | Index | 9.53% | -3.73% | 3.73% | 1 | 5% |
| **XAUUSD** | Commod | 8.47% | 4.94% | 4.94% | 0 | 0% |
| **Tesla** | Stock | -3.77% | -3.61% | 3.61% | 0 | 0% |
| **US NatGas Z8 (Z9)** | Commod | -6.96% | 13.09% | 13.09% | 1 | 18% |
| **JPYUSD** | CCY | -4.76% | 1.50% | 1.50% | 1 | 2% |
| **Coffee Z8 (Z9)** | Commod | -18.78% | -8.93% | 8.93% | 0 | 0% |
| **BTCUSD** | Crypto | 21.43% | -50.86% | 50.86% | 1 | 68% |
| **Wheat Z8 (Z9)** | Commod | 11.39% | -5.38% | 5.38% | 1 | 7% |
| **Total** |  | 3.43% | -67.41% |  | 5 | 100% |
| **Correlation** |  | **-51.32%** |  |  |  |  |

Table C.3: 1 year returns.

| **Underlying** | **Classification** | **Pre-Decision Return** | **Post-Decision Return** | **Absolute Post-Decision Return** | **Mean reverting** | **MR contribution** |
| --- | --- | --- | --- | --- | --- | --- |
| **EURUSD** | CCY | -5.57% | -4.29% | 4.29% | 0 | 0% |
| **Brent Z8 (Z9)** | Commod | -7.55% | -12.03% | 12.03% | 0 | 0% |
| **SPX TR** | Index | 9.53% | 8.40% | 8.40% | 0 | 0% |
| **XAUUSD** | Commod | 8.47% | 15.15% | 15.15% | 0 | 0% |
| **Tesla** | Stock | -3.77% | -17.67% | 17.67% | 0 | 0% |
| **US NatGas Z8 (Z9)** | Commod | -6.96% | -8.29% | 8.29% | 0 | 0% |
| **JPYUSD** | CCY | -4.76% | 3.45% | 3.45% | 1 | 23% |
| **Coffee Z8 (Z9)** | Commod | -18.78% | -14.08% | 14.08% | 0 | 0% |
| **BTCUSD** | Crypto | 21.43% | 43.24% | 43.24% | 0 | 0% |
| **Wheat Z8 (Z9)** | Commod | 11.39% | -11.42% | 11.42% | 1 | 77% |
| **Total** |  | 3.43% | 2.45% |  | 2 | 100% |
| **Correlation** |  | **75.52%** |  |  |  |  |
